# Quantifying High Resolution Transitional Breaks in Plant and Mammal Distributions at Regional Extent and Their Association with Climate, Topography and Geology

**DOI:** 10.1371/journal.pone.0059227

**Published:** 2013-04-01

**Authors:** Giovanni Di Virgilio, Shawn W. Laffan, Malte C. Ebach

**Affiliations:** 1 School of Biological Earth and Environmental Sciences, University of New South Wales, Sydney, NSW, Australia; 2 Australian Museum, Department of Palaeontology, Sydney, NSW, Australia; University of Kent, United Kingdom

## Abstract

**Objectives:**

We quantify spatial turnover in communities of 1939 plant and 59 mammal species at 2.5 km resolution across a topographically heterogeneous region in south-eastern Australia to identify distributional breaks and low turnover zones where multiple species distributions overlap. Environmental turnover is measured to determine how climate, topography and geology influence biotic turnover differently across a variety of biogeographic breaks and overlaps. We identify the genera driving turnover and confirm the versatility of this approach across spatial scales and locations.

**Methods:**

Directional moving window analyses, rotated through 360°, were used to measure spatial turnover variation in different directions between gridded cells containing georeferenced plant and mammal occurrences and environmental variables. Generalised linear models were used to compare taxic turnover results with equivalent analyses for geology, regolith weathering, elevation, slope, solar radiation, annual precipitation and annual mean temperature, both uniformly across the entire study area and by stratifying it into zones of high and low turnover. Identified breaks and transitions were compared to a conservation bioregionalisation framework widely used in Australia.

**Results/Significance:**

Detailed delineations of plant and mammal turnover zones with gradational boundaries denoted subtle variation in species assemblages. Turnover patterns often diverged from bioregion boundaries, though plant turnover adhered most closely. A prominent break zone contained either comparable or greater numbers of unique genera than adjacent overlaps, but these were concentrated in a small subsection relatively under-protected by conservation reserves. The environmental correlates of biotic turnover varied for different turnover zones in different subsections of the study area. Topography and temperature showed much stronger relationships with plant turnover in a topographically complex overlap, relative to a lowland overlap where weathering was most predictive. This method can quantify transitional turnover patterns from small to broad extents, at different resolutions for any location, and complements broad-scale bioregionalisation schemes in conservation planning.

## Introduction

Biogeographic breaks and transition zones have received considerable investigation at global and continental scales at coarse resolutions (e.g. [1]–[4]). Their detailed characterisation for distributions of multiple plant and animal species at smaller extents is less common. Some recent studies have adopted a narrower geographic focus, for instance Thiel-Egenter et al. [5] used a cluster analysis of plant species across the European Alps to assess the robustness of break zones, but this was at a comparatively coarse resolution.

It is increasingly pertinent to examine biogeographic phenomena at finer resolutions and at regional to local scales. This tests the generality of concepts derived by investigations over broad extents, as patterns and processes may not exhibit scale invariance [6], [7]. Practical concerns also advocate for translating insights achieved at broad scales to smaller extents. For instance, climatic effects vary over relatively small distances under topographic influence [8] with implications for how taxa respond to these changes. The effects of global processes such as climate change will ultimately need to be managed at regional and local scales [9], so it is important to understand how fine-scale variation in landscape features mediates climatic changes, particularly in areas of rich biodiversity.

Fine-scale quantification of biogeographic breaks also enables depiction of transitional zones in greater detail, facilitating their comparison with subtle variation in environmental gradients and potentially clarifying how different physical factors regulate species distributions across break transitions. This is useful for conservation planning because taxa occurring in transitional areas are often the most adaptive to a range of environmental conditions [10], [11]. Identifying continuous, fine-scale biogeographic distributional breaks may also promote design of efficient reserve networks. For instance, Shriner et al. [12] found that the numbers of species represented in equal area reserve networks increased as the grain size of distributional data decreased, such that best representation networks developed at coarser grains were almost 1000 times larger than equivalent networks using fine grained data. Fine resolution data may also be more informative to conservation planning in heterogeneous and fragmented landscapes [13] and in areas of high biological diversity [14], [15].

Quantifying the rate of species turnover across a landscape has been used to identify transitional turnover patterns and biogeographic breaks for bees [1] and birds [16]. Species turnover is the rate of compositional change in biotic communities across geographic space, i.e. the replacement of certain species with others among areas of any given size [17], [18]. High turnover areas experience greater rates of replacement between different species, and consequently show lower beta diversity and species richness, manifesting as a ‘break’ in distributions. Low turnover regions experience lower rates of community change and comprise more stable communities where the distributions of multiple different species overlap. Relating gridded maps of biotic turnover with corresponding environmental turnover can determine the relative influence of different physical factors on species turnover, and thus break formation. This was the general approach used by Di Virgilio et al. [19] to quantify breaks in plant, mammal and reptile distributions at 5 km resolution and their correspondence with geological and elevational turnover over a large portion of south-eastern Australia.

This research maps breaks and transitions in multiple plant and mammal species distributions at 2.5 km resolution across the south-east coast of New South Wales (NSW) in Australia, which is a smaller subsection of the same region previously examined by Di Virgilio et al. [19]. We provide a more detailed quantification of gradational breaks and overlaps in this region than was achieved by Di Virgilio et al. [19]. Break and overlap zones are also characterised more fully by identifying the specific flora and fauna exclusive to each zone and that may contribute to driving turnover. In changing the extent and resolution of these analyses, we also assess the versatility of this approach across multiple scales and resolutions and its potential for investigating transitional breaks at any location globally.

Secondly, Di Virgilio et al. [19] only examined inter-relationships between biotic and environmental factors uniformly across south-eastern NSW. Instead, we investigate whether the environmental influence on species turnover and break formation varies in different subsections of the study area, specifically in different geographic areas characterised by either high or low turnover. The environmental drivers considered are expanded to include climate (annual precipitation, annual mean temperature), topography (slope, solar radiation) and regolith weathering intensity, in addition to elevation and lithology. Some studies have identified climate as opposed to topographic variation as the principal driver of beta diversity in forest trees at a regional scale (e.g. [20]), whereas others have found a joint effect of climate and topography in regulating inter-annual vegetation variability [21]. We assess how the relative influence of environmental drivers varies for the different break and overlap zones.

Previous studies of trees in south-eastern NSW have shown the importance of variables such as climate, lithology and topographic position in predicting the richness of several species groups, e.g. [22], and how the environmental niche of eucalypt species varies along gradients of similar variables [23], [24]. The focus of our study is different in that it explicitly identifies transitional biogeographic breaks and overlaps for a greater variety of plant species and the role of spatial turnover of physical variables in driving these specific patterns.

Our results are interpreted in reference to the Interim Biogeographic Regionalisation for Australia version 6.1 (IBRA 6.1) [25], which is an existing bioregionalisation scheme widely used for conservation and reserve network planning in Australia [26], [27].

## Materials and Methods

### Study Area and Data Sources

**Figure 1:**
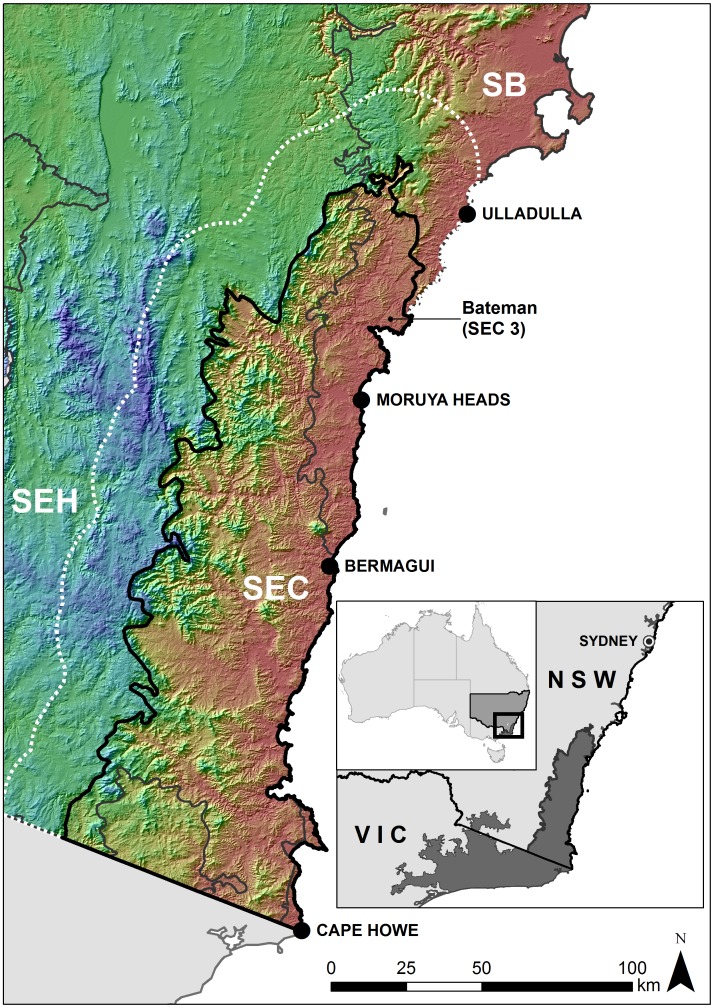
Location of the study area in south-eastern New South Wales, south-eastern Australia. Inset map shows the location of the South East Corner (SEC) Interim Biogeographic Regionalisation of Australia (IBRA) bioregion, which straddles New South Wales (NSW) and Victoria (VIC) in south-eastern Australia. Main map shows the part of SEC in coastal NSW that comprises the study area and its neighbouring IBRA bioregions in NSW (South-Eastern Highlands – SEH; Sydney Basin - SB). The white dashed line shows a 0.2° buffer which captured biotic and abiotic data around SEC in NSW for inclusion in moving window analyses of turnover.

The South East Corner (SEC) bioregion defined in IBRA 6.1 [25] straddles south-eastern New South Wales (NSW) and Victoria (VIC) in south-eastern Australia (Fig. 1). SEC in NSW comprises two parts: a montane region in the south-west and a larger area bordered by the Pacific Ocean, the South-Eastern Highlands (SEH) and Sydney Basin (SB) IBRA bioregions. We focused on SEC’s coastal portion in NSW (10,500 km^2^ in area) which is bordered by Cape Howe (149.974°, −37.494°) to the south and Ulladulla (150.467°, −35.358°) near its northern extent. SEC is further subdivided into three IBRA subregions.

SEC has a mesothermal climate, with long mild summers and little variation in annual rainfall. Average monthly temperatures range from minima of −3.5–8.4°C to maxima of 19.2–28.8°C. Average monthly rainfall minima and maxima are 29–102 mm and 58–155 mm respectively. SEC’s terrain is heterogeneous, comprising coastal lowlands extending ∼15 km inland to the southern highlands to the west which rise to ∼1250 m.

A 0.2° (∼22 km) buffer was added around SEC (dashed white line in Fig. 1) to minimise edge effects, as data just outside the SEC boundary would influence the compositional dissimilarities between species and environmental values some distance inside the border. The buffer did not extend into Victoria because the biotic data had been sampled mainly within NSW. Biotic and environmental data contained within this buffer are included in moving window analyses, but excluded from generalised linear model (GLM) analyses. The buffer increased the study area to 17,240 km^2^.

The geographic locations of plants and mammals sampled within SEC plus the buffer were derived from the NSW Office of Environment and Heritage’s (OEH) Atlas of NSW Wildlife [28] (see http://www.bionet.nsw.gov.au/). This database records the geographic location of flora and fauna sightings in NSW from 1788 to 2009. Some records showed multiple observations of the same species at the same location and time; these were treated as a single point. Exotic species were excluded and records identified as potentially ambiguous e.g. with duplicate names, subspecies where a parent species was present etc., were omitted. The remaining plant genera were checked for taxonomic accuracy, with no errors found. Marine mammals (order Cetacea and families Otariidae and Phocidae) were excluded because they are restricted to the coastal zone.

The plant database contained 104,030 records, comprising 145 families, 595 genera and 1939 species and included a wide variety of different trees, shrubs, ferns, tussocks, herbs, grasses and other flowering plants. There were 41,392 mammal records (38 genera, 59 species). The mean positional accuracy of the unaltered Atlas mammal data is 1265 m with a standard deviation of 3391 m. Plant observations have a mean accuracy of 1137 m and standard deviation of 3451 m.

Elevation data was derived from the three arc-second Shuttle Radar Topography Mission (SRTM) Digital Elevation Model (DEM) [29]. Slope and radiation data sets were derived from this DEM. Geological data were derived from the 1∶1,000,000 scale surface geology of Australia GIS dataset [30]. Lithological classes were simplified using the same recoding scheme as Di Virgilio et al. [19]. Annual precipitation and annual mean temperature at 30 arc-seconds (∼1 km) resolution were derived from the Worldclim data sets generated by Hijmans et al. [31]. Weathering intensity data were derived from the 100 m resolution weathering intensity index for Australia [32] which reflects changes to the physical characteristics of regolith as bedrock is altered into secondary minerals.

### Statistical Analyses

The spatial analyses adapted those described by Di Virgilio et al. [19] and used the Biodiverse software, version 0.16 [33]. The overall approach was to quantify biotic and environmental turnover by progressively iterating directional moving windows throughout the study area to measure the dissimilarity between two sets of cells, where each cell recorded the number of unique occurrences of species or environmental values at its geographic location. Window dimensions were calibrated using correlograms of biotic turnover versus distance. Correlograms are widely used to investigate variation in natural phenomena across geographic space, for example genetic structure [34] and plant richness gradients [35].

All biotic and abiotic data were aggregated to 0.0225°×0.0225° cells (∼2.5 km×2.5 km) by importing each data set into Biodiverse separately. All cells across the study area were used for the analyses, including those in which no species were recorded.

The Sørensen index was used to quantify the spatial turnover of biotic and lithological data:
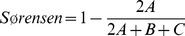
(1)where A is the number of unique occurrences of species or lithological classes found in both samples (i.e. both ‘neighbour sets’ of cells in the moving window); B is the number of species/classes unique to sample set one; and C is the number of species/classes unique to sample set two. This index produces a value between 0 (samples are identical) and 1 (samples share no species/classes). Alternative indices (Bray Curtis, Simpson’s Beta and Jaccard) were tested with the biotic data. The Jaccard index produced results similar to those of the Sørensen index, owing to the similarity in their calculation. Simpson’s Beta and Bray Curtis produced turnover patterns that shared some similarities with Sørensen-derived patterns, but were less detailed.

To investigate which genera are exclusive to discrete areas of high or low turnover, we identified the plant and mammal genera unique to each sample set and common to both. Once gradational areas of high and low plant turnover were identified via the moving window analyses, the outline of a discrete, high turnover ‘break’ zone was assigned to the sample one set and the perimeter of one of two discrete low turnover ‘overlap’ zones was assigned to the sample two set. The plant or mammal genera common to both sets and those exclusive to either sample set one or sample set two were then identified by comparing the genera in the break with those in one of the overlap areas.

Since the topographic, climatic and weathering variables are interval scaled data, the moving window analyses used a numeric dissimilarity index (eq. 2), which compared the set of numeric values in each sample set by calculating the mean absolute difference between all pairs of values in samples 1 and 2
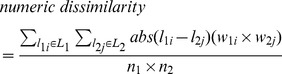
(2)where L_1_ and L_2_ are the sets of labels (values) in samples 1 and 2, respectively, and n_1_ and n_2_ are the overall abundances of labels in sample sets 1 and 2. w_1i_ and w_2j_ are the abundances of labels l_1i_ and l_2j_ in samples 1 and 2, respectively. This metric results in values ranging from zero (samples are identical) progressing theoretically to infinity as average differences increase.

We also calculated species richness and sample redundancy (eq. 3) for plants and mammals for each moving window. Richness is an important component of species turnover [36]. Sample redundancy [37] is a measure of sampling effort across SEC, and therefore enables reliability assessment (i.e. redundancy patterns can be reconciled against species turnover patterns in order to gauge the effects of poor sampling), and was calculated as:
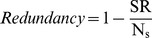
(3)where SR is the species richness and N_s_ is the number of observations of each species in the relevant sample set. This equation produces values from 1 in cases where there are many observations for each species and indicating areas that are well sampled, to zero, meaning there is no redundancy in the sampling because there is only one observation per species.

### Moving Window Analyses

The mosaic of cells comprising a moving window were divided into one of two orthogonal, semi-elliptical neighbourhood sets (the sample one and two sets described above) and used the same geometric specification as the moving windows described by Di Virgilio et al. [19]. However, in this study the neighbourhood sets used different dimensions, calibrated via correlograms using the 0.0225° resolution biotic data.

The same window sizes were used for flora and mammals to aid comparison of turnover in each group. Since we aim to find the environmental correlates of species turnover, the same dimensions were used for moving window analyses of the environmental variables.

To gauge how biotic and abiotic turnover varies in different directions, moving window analyses were repeated in 25 increments of 15°; hence the window was rotated through 24 different angles from 0° to 360°.

### Generalised Linear Model Regression

We investigated how environmental variables influenced species turnover across the whole study area and also how their relative influence on break and overlap formation varied once the study area was stratified into three different areas of high or low turnover (see Results for details of the zones/stratification). Generalized linear models (GLM; [38]) were used to regress untransformed plant or mammal turnover on the separate environmental turnover predictors generated by each of the 25 moving window orientations for the complete and stratified study area. A Gaussian error distribution for biotic turnover was assumed. We opted for univariate models because a multivariate approach may not capture important covariates.

## Results

### Correlograms

**Figure 2:**
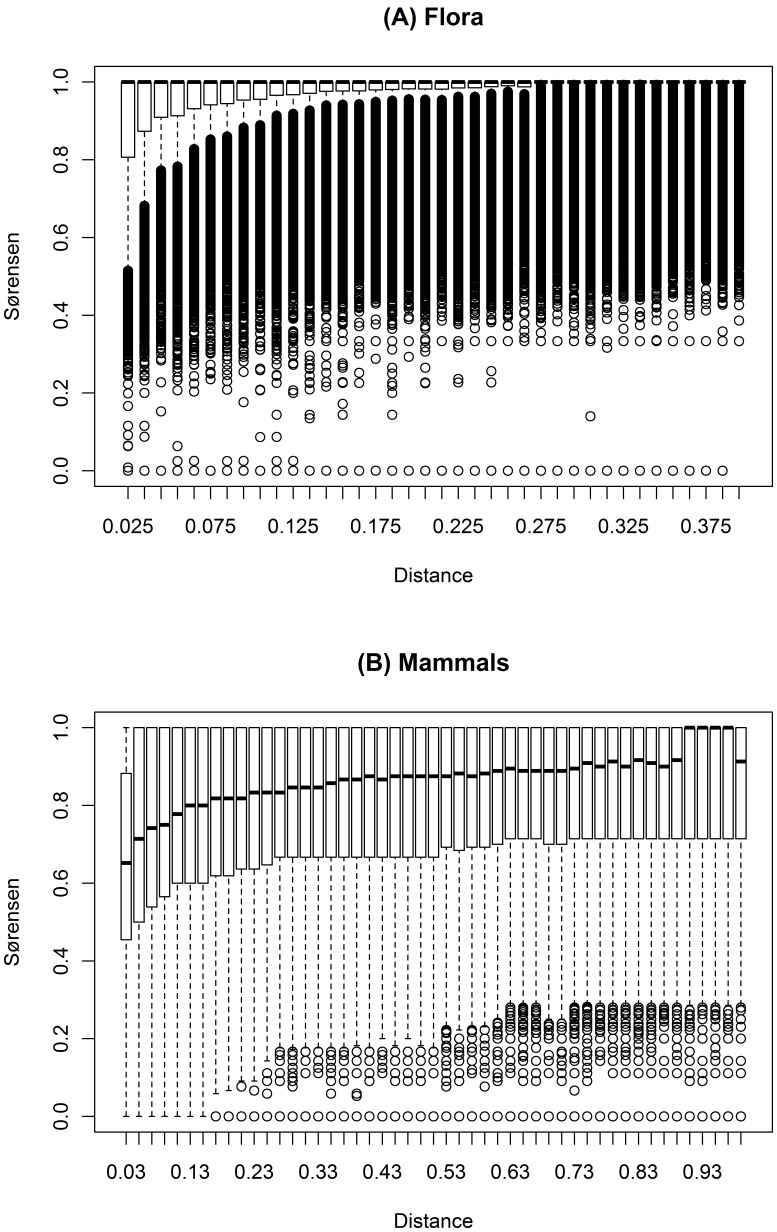
Species group correlograms showing the distribution of turnover values between pairs of cells over increasing geographic distance. The species group correlograms for (A) flora and (B) mammal turnover which were used to calibrate the dimensions of moving window analyses of biotic and abiotic compositional dissimilarity. The correlograms are depicted as boxplots showing the distribution of turnover values between pairs of cells over geographic distance. Hence, for each boxplot, the black horizontal bar denotes the median turnover, the top and bottom of each box represent the 75th and 25th percentiles, respectively. The whiskers represent the minimum and maximum of all the data at each distance. Circles represent outliers. The distance on the x-axis at which the median turnover values begin to plateau is the maximum distance to which there is spatially structured turnover.

The correlograms revealed a range of ∼0.2° for flora, with a sill value of ∼0.92, where the range is the distance on the x-axis at which the median turnover values begin to plateau and is the maximum distance to which there is spatially structured turnover (Fig. 2A). The mammal data had a range of ∼0.25° and a sill value of ∼0.8 (Fig. 2B). Therefore, the moving window outer ellipse had major and minor radii of 0.2° and 0.085° respectively. The inner ellipse major and minor radii were 0.085° and 0.0075°.

### Species Richness

**Figure 3:**
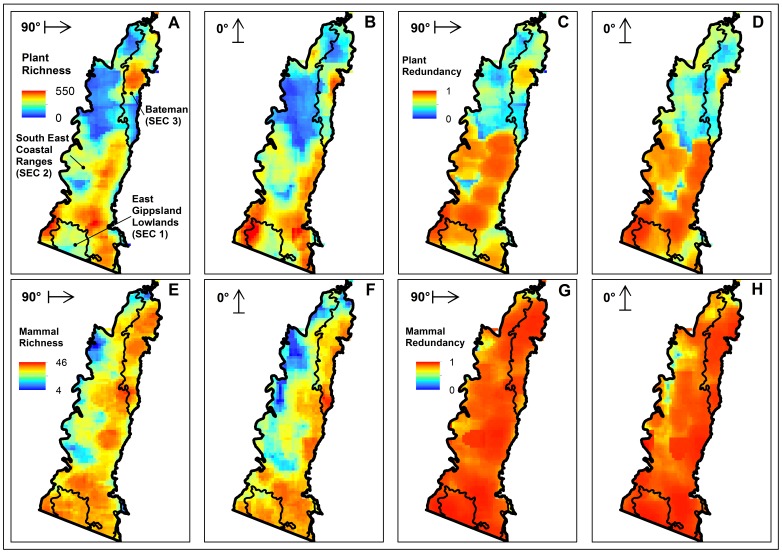
Plant and mammal richness and sample redundancy patterns. Plant (panels A–D) and mammal (panels E–H) richness and sample redundancy patterns in the South East Corner (SEC) bioregion of south-eastern New South Wales, Australia, for the 90° and 0° window orientation analyses. Panel A labels the main Interim Biogeographic Regionalisation of Australia (IBRA) subregions of SEC. The arrows (not drawn to scale) in the top left corner of each panel represent the overall orientation of the moving window used for that panel. Note that the arrow base shows the orientation of neighbour set 1, with the arrow indicating the direction of neighbour set 2.

Plant richness was high across much of the SEC bioregion (Fig. 3, panels A–B), except for a large area of low richness in the north. Patches of higher plant richness tended not to extend across IBRA boundaries, e.g. the East Gippsland Lowlands (SEC 1). Most high richness areas were on low, uniform terrain, especially along the coast, except for a patch of high richness in the southwest.

A greater proportion of SEC showed high mammal species richness (Fig. 3, panels E–F) with only small patches of low richness restricted to higher elevations. Nonetheless, some areas of high mammal richness corresponded with distinct regions of high plant richness e.g. the coastal lowlands from southern Bateman (SEC 3) to SEC’s southern border.

Richness patterns for both biotic groups changed slightly according to the orientation of the moving window because different combinations of cells are considered by different window orientations. For example, the shape of the northern low plant and mammal richness zones changed from the 90° to 0° windows, but their overall form was still consistent across orientations.

### Sample Redundancy

Floristic sampling redundancy (Fig. 3, panels C–D) generally resembled plant richness patterns, with an area of medium to lower redundancy roughly corresponding to the northern low richness zone. High sampling redundancy extended from the southern Bateman bioregion to SEC’s southern border, interspersed with only a few patches of low redundancy. In comparison to plants, mammal redundancy patterns showed less variation with higher redundancy across a larger portion of SEC.

### Biotic Turnover

**Figure 4:**
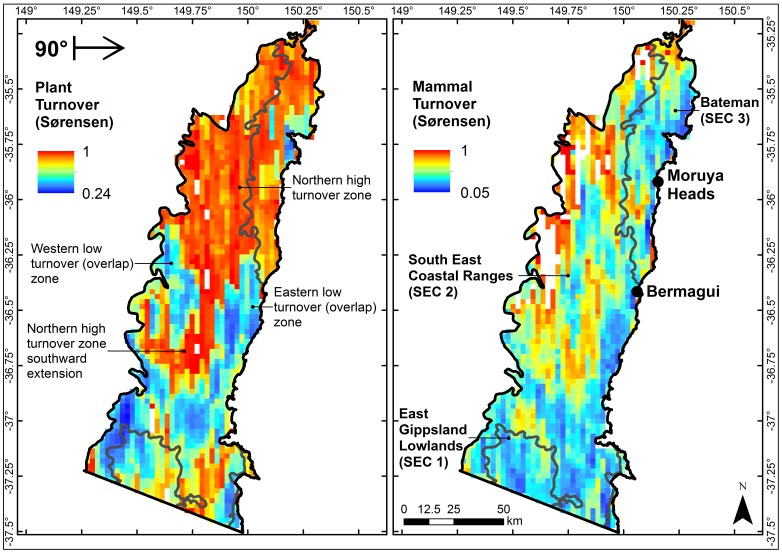
Plant and mammal species turnover maps showing location of turnover zones. Plant and mammal species turnover maps in the South East Corner (SEC) bioregion of south-eastern New South Wales, Australia, for the Sørensen moving window analyses at bearing 90°. The plant turnover map labels discrete zones of high and low turnover. The mammal turnover map also shows the Interim Biogeographic Regionalisation of Australia (IBRA) subregions comprising this part of SEC and some of the principal towns.

Figure 4 shows the biotic turnover results for moving window analyses oriented at 90°. White areas on the maps are where either one or both of the moving window neighbourhoods contain no observations.

#### Flora

There was continuous variation in species turnover patterns with several breaks, overlaps and transition zones shown in greater detail than the 5 km resolution analyses of Di Virgilio et al. [19]. A prominent area of high plant turnover spanned northern SEC, with a strip of high turnover extending southward. The main body of this ‘northern break zone’ straddled a topographically complex area to the west, but flatter terrain along the coast. The southward extension corresponds to a low, flat area with undulating terrain along its southern boundary. Much of this break shows comparatively uniform turnover, although there is greater variation along its edges where there are transitions from high to low turnover in the south-east and south-west. Although Di Virgilio et al. [19] identified the location of this floristic break zone, our results show this break in much greater detail, with the presence of the southern extension and transitions along the zone boundaries more apparent.

A western low turnover area to the south-west of the break overlies high, topographically complex terrain separated from the break by a small valley (Fig. 1). In contrast, there is an eastern low turnover zone situated on the coastal plain. The breaks and overlaps varied in shape with moving window orientation, but again their overall form remained consistent (see Figures S1, S2, S3).

**Figure 5:**
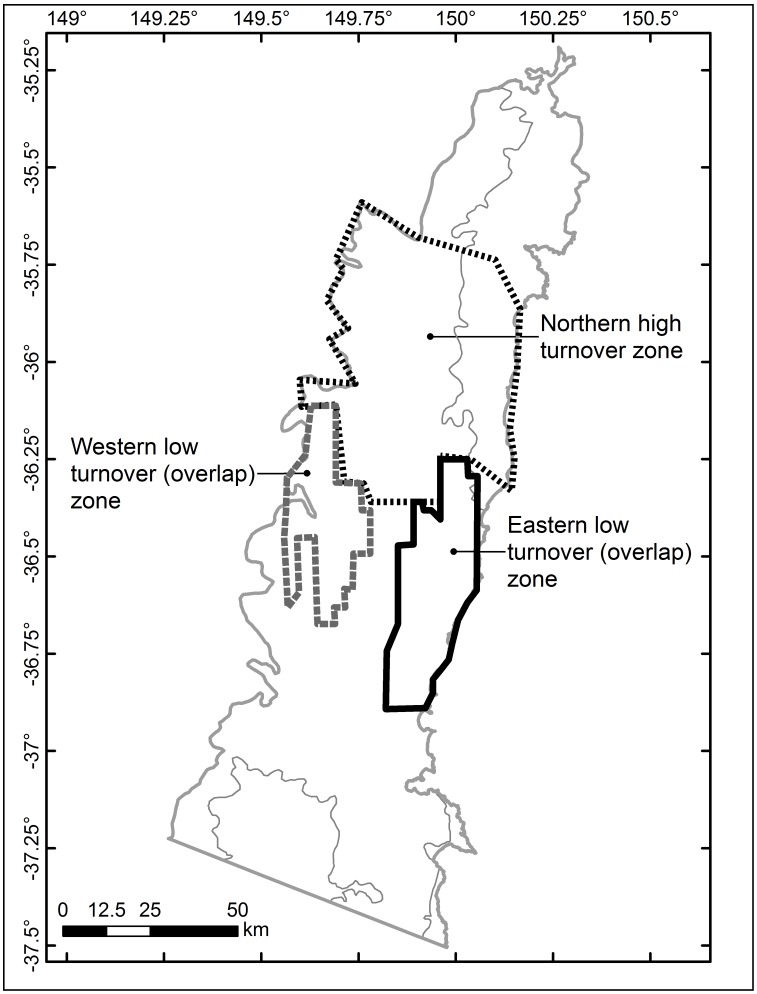
Discrete high and low plant turnover zones in the study area. Approximate extents of a discrete high plant turnover zone (‘Northern high turnover zone’, black dotted line) and low plant turnover zones (‘Eastern low turnover zone’, black solid line and ‘Western low turnover zone’, grey dashed line) identified via moving window analyses of plant turnover in the South East Corner (SEC) bioregion of south-eastern New South Wales, Australia.

The outlines of the northern break zone, the hilly western overlap and the coastal eastern overlap are sketched in Fig. 5 and form the basis of the stratification of SEC into discrete turnover zones and the identification of genera exclusive to each zone. Although other discrete low turnover zones were present in SEC, these two overlaps were specifically selected because they flank the northern break on landscapes of varying topographic complexity.

#### Mammals

Mammal turnover was lower across SEC and less sharply defined than plant turnover (Fig. 4), but there were some parallels with plant turnover patterns. Parts of the northern floristic break were evident, such as the western and north-eastern edges and southward extension, although all were fainter and smoother. Most of the coastline showed low mammal turnover, whereas high plant turnover extended from northern SEC to southern Bateman. Overall, mammal turnover adheres less to IBRA boundaries than plants, although some mammal turnover patterns do not extend across the boundaries such as the high turnover that aligns with the East Gippsland Lowlands subregion.

Biotic turnover was not as smooth as the corresponding richness and redundancy patterns. Breaks in mammal distributions appeared in both high and low redundancy areas and across extremes of richness. In contrast, the main portion of the floristic high turnover zone corresponds to areas of low to medium sampling redundancy and richness.

#### Genera driving high turnover

The northern break zone and the smaller eastern overlap contained similar numbers of plant species and genera, although eight times as many observations were sampled in the overlap (Table 1). 79 plant genera occurred exclusively within each zone, with 270 genera common to both regions (Table 2). Most of the 270 common genera are widespread throughout SEC. However, of the 79 genera exclusive to the break, 14 occur only in the break and nowhere else within SEC, while 45 genera are isolated from other populations elsewhere within SEC. The eastern overlap shows a similar trend: 11 genera occur nowhere else and 35 are isolated subpopulations.

**Table 1:** Total number of plant and mammal observations, genera and species sampled within the northern biogeographic break zone, eastern overlap and western overlap within the South East Corner (SEC) study area in New South Wales, Australia.

Turnover Zone	No. Observations	No. Genera	No. Species
**Northern Break**			
Plants	2385	348	740
Mammals	3961	34	47
**Eastern Overlap**			
Plants	18610	334	701
Mammals	3874	33	46
**Western Overlap**			
Plants	5803	265	521
Mammals	349	26	33

**Table 2:** The number of plant and mammal genera that occur exclusively either in the northern biogeographic break zone, or eastern overlap, and common to both areas, compared with the number of plant and mammal genera that occur exclusively either in the northern break zone, or western overlap and genera common to both areas, within the South East Corner (SEC) study area in New South Wales, Australia.

Species Group	No. Genera Common to BothRegions (Both Neighbour Sets:List A Genera)	No. Genera Unique to Break (Neighbour Set 1: List B Genera)	No. Genera Unique to Overlap (Neighbour Set 2: List C Genera)
**Break v Eastern overlap**			
Plants	270	79	79
Mammals	32	2	1
**Break v Western overlap**			
Plants	214	135	57
Mammals	26	8	0

A greater variety of plant genera and species were sampled in the northern break relative to the western overlap (Table 1). There were 135 genera unique to the break, 57 unique occurrences in the western overlap and 214 common genera (Table 2). The distribution of unique/common genera showed a similar pattern to that noted above. Plant genera identified by all comparisons are listed in Tables S1–S2 in File S1.

Similar numbers of mammal observations, genera and species were sampled in the break and eastern overlap (Table 1). There were two genera unique to the break, one unique genus in the overlap and 32 genera common to both areas (Table 2). Comparison of the break and western overlap revealed 8 genera unique to the former, whilst the overlap contained no exclusive genera and 26 genera which occurred in both areas (Table 2). In contrast to plants, mammal genera unique to the break were commonplace throughout SEC. See Tables S3–S4 in File S1 for the mammal genera identified.

### Abiotic Turnover

**Figure 6:**
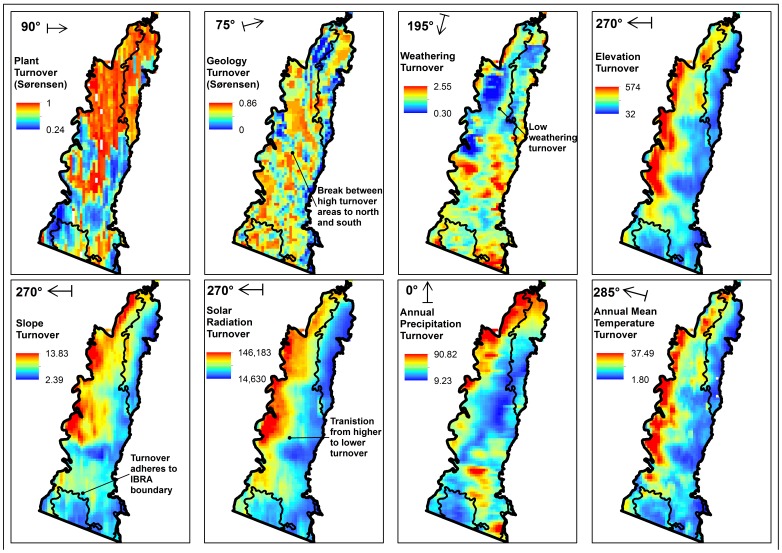
Plant species turnover compared to environmental turnover. Plant and abiotic turnover maps in the South East Corner (SEC) bioregion of south-eastern New South Wales, Australia, for the Sørensen and numeric dissimilarity moving window analyses at bearings showing the strongest correlative relationship between environmental and plant turnover. Turnover maps show Interim Biogeographic Regionalisation of Australia (IBRA) bioregion boundaries.

Generally, environmental turnover did not correspond to the prominent biotic turnover zones (Fig. 6), though there were some fine-scale parallels. For instance, slope and solar radiation show a southern transition of low turnover that corresponds to the southward extension of the northern floristic break. Nonetheless, concordance between biotic and abiotic turnover patterns was inconsistent. The western plant overlap is on high elevation, topographically complex terrain showing higher elevation turnover, whilst the opposite applies to the eastern overlap. Elevation, slope, radiation and annual precipitation show substantial patches of higher turnover in north-western SEC, corresponding to part of the biotic northern break, whereas weathering turnover is low in this same area. With the exception of geology and weathering, abiotic turnover is low along the majority of the coastal lowlands.

### Generalised Linear Models of Biotic and Abiotic Turnover

#### Turnover across the complete study area

Topographic and temperature turnover was substantially more predictive of variation in mammal turnover than for plants, as shown by the maximal *R^2^* coefficients in Table 3. In contrast, weathering and geological turnover were poor predictors of both mammal and plant turnover, explaining only a small proportion of variation in biotic turnover. Biotic-environmental turnover relationships were positive across SEC, except for weathering turnover. All maximal *R^2^* coefficients were significant at *P*<0.001.

**Table 3:** Maximum *R^2^* coefficient of determination, regression coefficient (β_1_) values and t-scores produced by generalised linear model regressions between the corresponding biotic and environmental turnover results generated by 25 moving window orientations iterated throughout the South East Corner (SEC) bioregion of south-eastern New South Wales, Australia.

Correlates				
Abiotic	Biotic	R^2^	β_1_	*t*
Geology	Flora	0.015	0.123	5.386
	Mammals	0.015	0.133	5.348
Weathering	Flora	0.029	−0.276	−7.584
	Mammals	0.023	−0.312	−6.931
Elevation	Flora	0.035	0.260	8.635
	Mammals	0.249	0.698	25.613
Slope	Flora	0.055	0.346	10.936
	Mammals	0.246	0.771	25.769
Solar Radiation	Flora	0.016	0.161	5.808
	Mammals	0.247	0.622	25.467
Annual Precipitation	Flora	0.028	0.322	7.766
	Mammals	0.068	0.469	12.023
Annual Mean Temperature	Flora	0.039	0.236	8.779
	Mammals	0.247	0.698	25.446

#### Geographically stratified turnover

GLM regressions between biotic and environmental turnover in the stratified study area produced the maximal *R^2^* coefficients and corresponding regression coefficients shown in Table 4. The two *R^2^* coefficients in bold were not significant (flora-temperature *P* = 0.108; flora-elevation *P* = 0.026), whilst all other maximal coefficients were significant at *P*<0.01, with many having very small *P* values.

**Table 4:** Maximum *R^2^* coefficient of determination and regression coefficient (β_1_) values and t-scores produced by generalised linear model regressions between the corresponding biotic and environmental turnover patterns generated by 25 moving window orientations, when these results were stratified into a discrete break zone and adjacent eastern and western overlaps in the South East Corner (SEC) bioregion of south-eastern New South Wales, Australia.

Correlates		Northern Break Zone			Eastern Overlap			Western Overlap		
Abiotic	Biotic	R^2^	β_1_	*t*	R^2^	β_1_	*t*	R^2^	β_1_	*t*
Geology	Flora	0.084	0.070	6.467	0.041	0.268	2.762	0.099	0.504	3.848
	Mammals	0.093	0.438	6.875	0.077	0.267	3.851	0.070	0.297	2.706
Weathering	Flora	0.033	0.083	4.122	0.112	0.354	4.770	0.156	0.487	4.662
	Mammals	0.048	0.263	4.744	0.088	0.337	4.177	0.089	−*0.348*	−2.884
Elevation	Flora	0.014	0.069	2.673	**0.027**	−*0.253*	−*2.248*	0.183	−*0.524*	−*5.145*
	Mammals	0.288	0.826	13.667	0.288	0.800	8.510	0.076	0.399	3.194
Slope	Flora	0.073	0.107	6.333	0.066	−*0.399*	−*3.576*	0.189	0.519	*5.238*
	Mammals	0.245	0.696	12.238	0.280	0.662	8.333	0.175	0.709	4.557
Solar Radiation	Flora	0.027	0.059	3.743	0.096	−*0.319*	−*4.383*	0.106	−*0.410*	−*4.008*
	Mammals	0.261	0.647	12.753	0.223	0.610	7.185	0.141	0.483	4.519
Precipitation	Flora	0.034	0.092	4.184	0.080	0.435	3.947	0.076	0.405	3.347
	Mammals	0.196	0.633	10.422	0.160	0.653	5.833	0.128	0.576	4.276
Temperature	Flora	**0.031**	−*0.021*	−*1.612*	0.039	−*0.455*	−*2.714*	0.168	−*0.529*	−*4.877*
	Mammals	0.273	0.816	13.114	0.285	0.739	8.445	0.076	0.324	3.036

Several differences between the biotic-environmental relationships emerged within the different turnover zones. Topographic and climatic turnover were more strongly predictive of mammal turnover in the northern break and eastern overlap compared to the western overlap, whilst geology and weathering turnover showed comparatively weaker associations with mammal turnover across all three zones. In comparison to mammals, environmental turnover had limited predictive capacity for variation in plant turnover, except for in the western overlap where weathering, elevation, slope and temperature turnover were substantially more predictive. Weathering turnover was also much more predictive of plant turnover than the other variables in the eastern overlap.

Most regression slopes were positive, but there were some negative relationships between biotic and environmental turnover. These occurred mainly in the two overlap zones, with only one negative slope in the northern break, and applied most consistently to relationships between plant, elevation, solar radiation and temperature turnover.

## Discussion

This research aimed to identify high resolution biogeographic breaks and transitions in multi-taxon distributions, the specific genera driving break formation and the environmental role in generating these patterns using a study area in south-eastern Australia as an example. Biotic and environmental turnover patterns in this region were depicted in substantially greater detail than was achieved by Di Virgilio et al. [19]. This is due the more localised extent of the present analyses, their finer resolution and the smaller dimensions of the moving window analyses. Gradational variation in turnover within and along the boundaries of break and overlap zones was shown more clearly and denoted the continuous change in species assemblages. Other studies such as [1], [2], [5], [16] have shown variation in turnover along the length of biogeographical boundaries, but over broad extents and at coarse resolutions. This more detailed characterisation of transitions in biotic and environmental breaks and overlaps could be informative to conservation planning, since priority is often assigned to areas of ecological transition where multiple taxa from adjacent assemblages overlap [39]. Typically conservation strategy relies on broad-scale ecoregions (e.g. IBRA) with solid boundaries, the limitations of which have been raised by others, e.g. [40].

**Figure 7:**
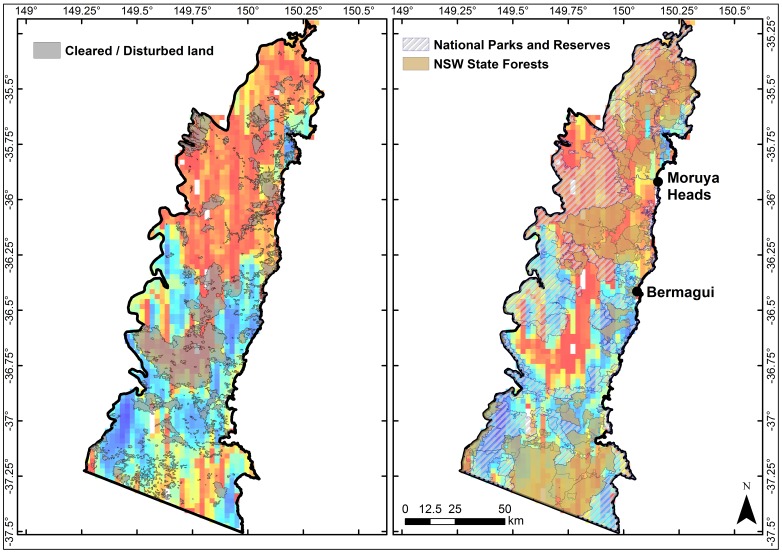
Plant species turnover overlaid with the location of cleared/disturbed land and reserves, national parks and state forests. Plant species turnover maps overlaid with the locations of cleared/disturbed land (left panel) and national parks, reserves and New South Wales (NSW) state forests (right panel).

The apparent breaks and overlaps do not appear closely linked to differing land-use patterns, except for an area of cleared/disturbed land which corresponds with the location of the southern extension of high plant turnover (Fig. 7). However, there are no further obvious parallels between cleared/disturbed land and the other main turnover patterns. National parks, reserves and NSW state forests are located in the same locations as the main portion of the northern break and each of the adjacent overlaps (Fig. 7). Since protected land and state forests exist in both high and low turnover areas, it seems unlikely that turnover patterns principally reflect land usage patterns. Moreover, the shape of the eastern overlap does not match the outline of the reserves and state forests.

We also quantified sample redundancy across SEC to reconcile the turnover patterns with the effects of poor sampling. Plant sampling redundancy ranges from low to medium levels in the north of the study area (Fig. 3, panels C and D), which is the approximate location of the large floral break zone, so it is unlikely that poor sampling alone explains the existence of this break. Redundancy variation is also smoother and more uniform than the turnover patterns, e.g. most of southern SEC shows uniformly high sample redundancy, but there are two disjunct areas of low turnover in this same area and not uniform, low turnover.

Despite some fine-scale similarities, overall the environmental turnover maps lacked consistent parallels with biotic turnover. For instance, high and low environmental turnover corresponded to separate areas of low biotic turnover. This appears to support the contention that greater environmental diversity may not always represent increased species richness [41].

Generalised linear model (GLM) regressions between biotic and abiotic turnover across SEC were much more predictive of mammal turnover than for plants. The positive relationships between most physical variables and biotic turnover suggest an intrinsic link between higher rates of environmental turnover and higher biotic turnover across SEC, although the opposite trend applied to weathering intensity. Relationships between plant and environmental turnover were weak across SEC, and varied only slightly. This contrasts with strong relationships between topography, temperature and mammal turnover, suggesting variation in these variables is influencing mammal turnover across SEC.

However, the *R^2^* maxima derived from GLM regression fitting when SEC was stratified into discrete high and low turnover zones provided more insight into the relative contribution of different physical factors in affecting turnover. For instance, topography and climate were more predictive of mammal turnover in the northern break and eastern overlap, but markedly less so in the western overlap. Previous studies at continental scale have shown a joint role for topographic complexity and climate in influencing mammal turnover [42], but these results at a regional extent show how these associations vary in different areas with different geographic and environmental characteristics. Conversely, topography and climate lacked strong relationships with plant turnover in the low, uniform landscape of the eastern overlap and the northern break zone, but they were much more predictive of plant turnover in the hilly western overlap, which is broadly consistent with some previous findings. For instance, the response of inter-annual vegetation variability to climatic changes was closely linked to topographic variability [21]. Species distribution models of *Eucalyptus fastigata* in south-eastern NSW provided misleading range estimates when using only climatic variables and omitting topography [43]. The western overlap also contained 39 *Eucalyptus* species compared to 29 species in the eastern overlap, which accords with the finding of Austin et al. [22] that eucalypt species richness is lower on flats and in gullies compared to on ridges and slopes.

Negative relationships between environmental and biotic turnover were more common to plants and were exclusive to the two low turnover zones, with the exception of plant-temperature turnover in the northern break, which was only slightly negative (β_1_ = −0.021). This suggests that greater environmental variability in the break influences higher biotic turnover and potentially lower overall beta diversity and species richness. In contrast, increased variability in elevation, slope, solar radiation and temperature within the low turnover zones is associated with lower plant turnover and hence higher species richness because the distributions of multiple species overlap in these regions. Complex, heterogeneous environments have often been associated with higher species richness because of the greater variety of habitats and more opportunities for resource exploitation that they provide [44]–[46]. The negative biotic-environmental relationships in the two overlap zones are broadly consistent with this axiom. However, the large number of positive relationships between biotic and environmental turnover, especially within the break zone, suggest the opposite pattern with greater environmental heterogeneity engendering higher species turnover and consequently lower richness.

The northern break zone and the adjacent, smaller eastern overlap both contained similar genera and species numbers, despite many more observations being sampled in the latter. This overlap contains several cells of particularly low turnover, plus a band of low turnover extending ∼25 km southwest and connecting with a separate low turnover zone at −37°. These low turnover cells indicate the presence of comparatively stable communities with high richness and diversity. They also coincide with areas of relatively low topographic and climatic variability. The connecting strip is 4 to 8 km in width and mostly comprises reserves and NSW state forests, though there is some incursion of cleared/disturbed land at its narrowest point. Nonetheless, this connecting strip of low turnover warrants investigation as a potential corridor linking the two overlaps, especially since corridors comprising natural landscape features may increase species movement in fragmented landscapes [47].

The eastern overlap may also be viable for additional conservation reserve siting. A key goal of conservation is to maximise biodiversity persistence [48], [49], which may be achieved by a variety of measures, such as maximising species abundances [50], minimising habitat fragmentation [51] and buffering against outside threats with compact reserve designs [52]. The eastern overlap appears to satisfy some of these criteria, especially since it contains a similar variety of genera and species to the much larger neighbouring break, but in a significantly smaller area, which may be more cost-effective. Currently this overlap hosts both dedicated nature reserves and NSW state forests, with the latter amenable to forestry operations regulated by the *Forestry and National Park Estate Act*, 1998 [53].

**Figure 8:**
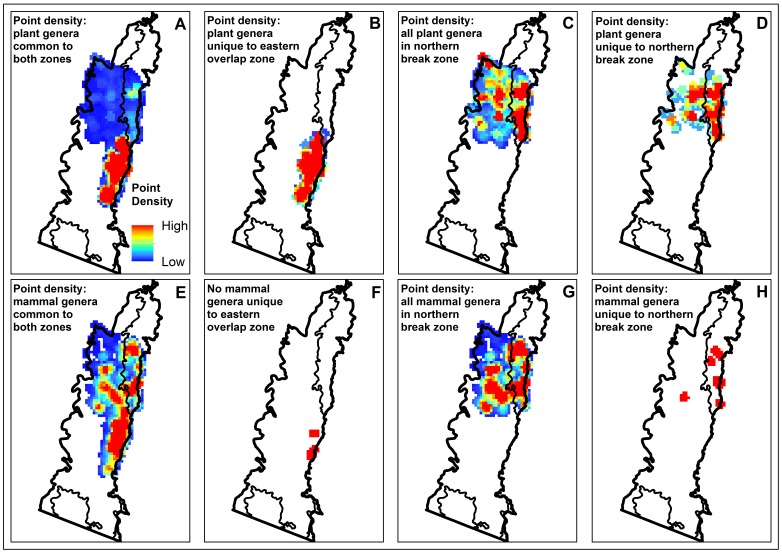
Distribution densities of plant and mammal genera unique and common to break and overlap zones. Relative distribution densities (red = high density; blue = low density) of plant and mammal genera point observations common to the northern break and eastern overlap zones (panels A and E), unique to the eastern overlap zone (panels B and F), all plant and mammal genera in the northern break zone (panels C and G) and plant and mammal genera unique to the break zone (panels D and H) in the South East Corner (SEC) region of south-eastern New South Wales, Australia.

The northern break zone contained comparatively high proportions of unique plant and mammal genera and these same genera may play a significant role in driving higher rates of turnover. Figure 8 (panel A) shows the distribution density of plant genera common to the break and eastern overlap, with the majority occurring in the overlap. Genera exclusive to the eastern overlap are evenly distributed (Fig. 8, panel B). Both findings are expected because this is an area of comparatively stable, high diversity. That the high turnover zone hosts more than twice the number of unique plant genera than the western overlap and the same number as the eastern overlap is interesting, since break zones are characterised by higher rates of community change and lower overall diversity. However, the majority of these unique genera are clustered in a small portion of the break, in the coastal strip within the southern part of the Bateman bioregion (Fig. 8, panel D). This section of the Bateman bioregion has relatively low environmental turnover, but this increases further inland in the break zone where there are no exclusive genera. Plant genera sampled in the break are evenly distributed (Fig. 8, panel C), hence this pattern is not simply due to biased sampling.

The distribution of mammal genera in the break and eastern overlap zones shows the same general pattern (Fig. 8 panel E) with more unique genera occurring exclusively in the break and also clustering in the southern part of the Bateman bioregion (Fig. 8, panel H). Although there are several state forests in south Bateman, ∼11% of this region is protected by reserves (Fig. 7), hence this area may also warrant consideration in conservation planning.

### Conclusions

We have mapped gradational biotic and environmental turnover patterns in significantly greater detail and for a smaller region than Di Virgilio et al. [19], demonstrating the applicability of this method to a range of scales and resolutions, potentially for any global location. There was a combination of concordance and divergence between species turnover patterns and the widely used IBRA bioregionalisation framework, which cautions against applying this framework too rigidly in natural resource planning. The environmental influence on turnover differed for mammals and plants in different subsections of SEC corresponding to separate break and overlap zones. For instance, topographic and temperature turnover showed stronger associations with plant turnover in a hilly, high altitude overlap zone compared to a homogenous coastal overlap. The capacity to quantify the gradational variation of discrete break and overlap zones, depict their transitional boundaries in high resolution, and identify genera exclusive to each zone could inform conservation planning, for instance by identifying candidate reserve areas.

## Supporting Information

Figure S1 Plant species turnover maps in the South East Corner (SEC) bioregion of south-eastern New South Wales, Australia, for Sørensen moving window analyses rotated through 360° in 15° increments. Plant species turnover maps, moving window orientations 90° to 315°.
(TIF)

Figure S2 Plant species turnover maps in the South East Corner (SEC) bioregion of south-eastern New South Wales, Australia, for Sørensen moving window analyses rotated through 360° in 15° increments. Plant species turnover maps, moving window orientations 300° to 165°.
(TIF)

Figure S3 Plant species turnover maps in the South East Corner (SEC) bioregion of south-eastern New South Wales, Australia, for Sørensen moving window analyses rotated through 360° in 15° increments. Plant species turnover maps, moving window orientations 150° to 90°.
(TIF)

File S1 Comparison of the plant and mammal genera unique to northern break and eastern overlap zones, unique to the same break and a western overlap zone and common to the intersections of each pair. **Table S1,** Plant genera that occur exclusively in the northern biogeographic break zone (n = 79), exclusively in the eastern overlap zone (n = 79) and genera that occur in both areas (n = 270) within the South East Corner (SEC) study area in New South Wales, Australia. **Table S2,** Plant genera that occur exclusively in the northern biogeographic break zone (n = 135), exclusively in the western overlap zone (n = 57) and genera that occur in both areas (n = 214) within the South East Corner (SEC) study area in New South Wales, Australia. **Table S3,** Mammal genera that occur exclusively in the northern biogeographic break zone (n = 2), exclusively in the eastern overlap zone (n = 1) and genera that occur in both areas (n = 32) within the South East Corner (SEC) study area in New South Wales, Australia. **Table S4,** Mammal genera that occur exclusively in the northern biogeographic break zone (n = 8), exclusively in the western overlap zone (n = 0) and genera that occur in both areas (n = 26) within the South East Corner (SEC) study area in New South Wales, Australia.
(DOC)
