# Supplementary figures and images for: Quantifying High Resolution Transitional Breaks in Plant and Mammal Distributions at Regional Extent and Their Association with Climate, Topography and Geology
Source: PLoS One. 2013 Apr 1;8(4):e59227. doi: 10.1371/journal.pone.0059227 (PMC3613380; doi:10.1371/journal.pone.0059227)

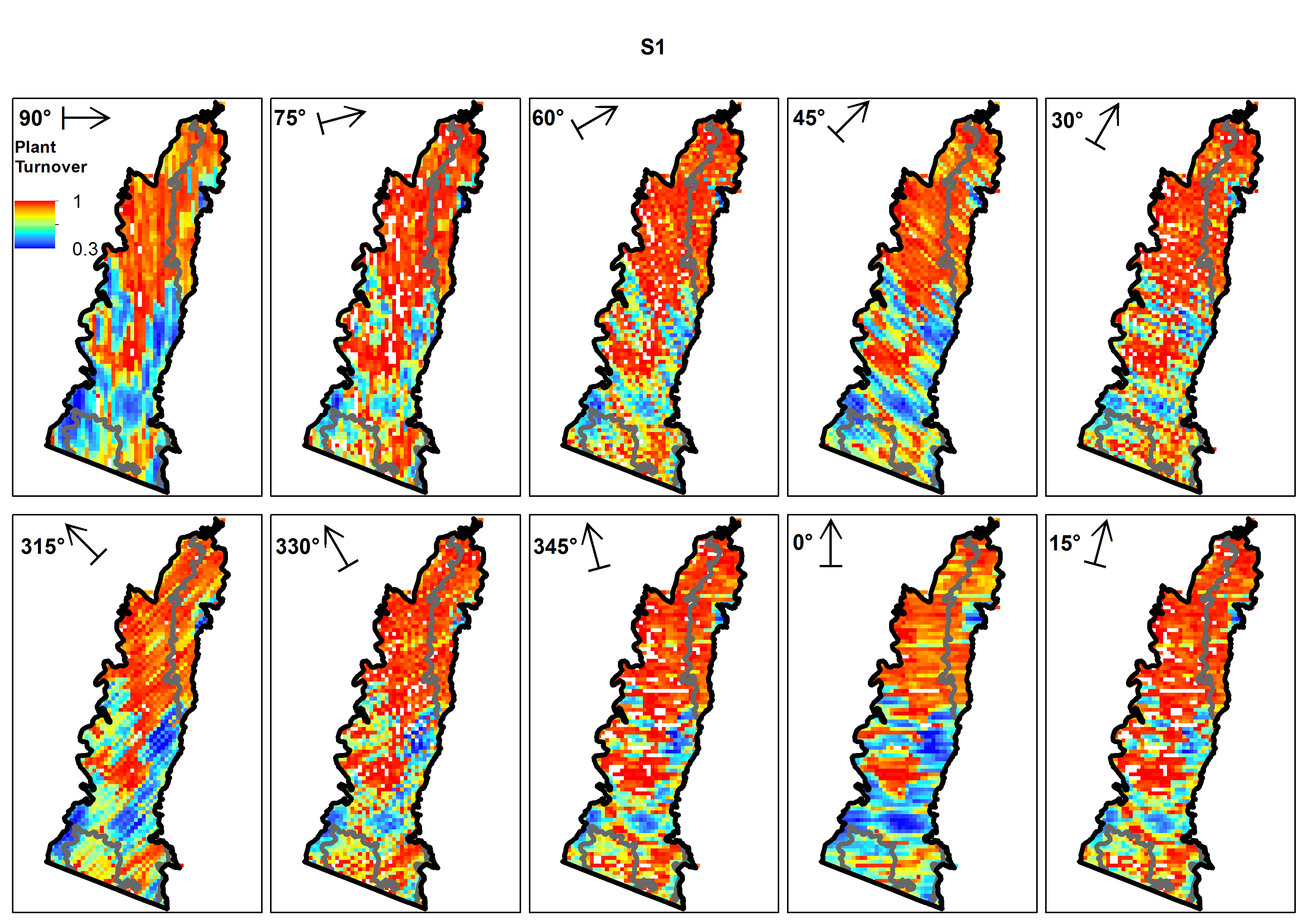

Supplement: Figure S1 — Plant species turnover maps in the South East Corner (SEC) bioregion of south-eastern New South Wales, Australia, for Sørensen moving window analyses rotated through 360° in 15° increments. Plant species turnover maps, moving window orientations 90° to 315°. (TIF) [file pone.0059227.s001.tif]

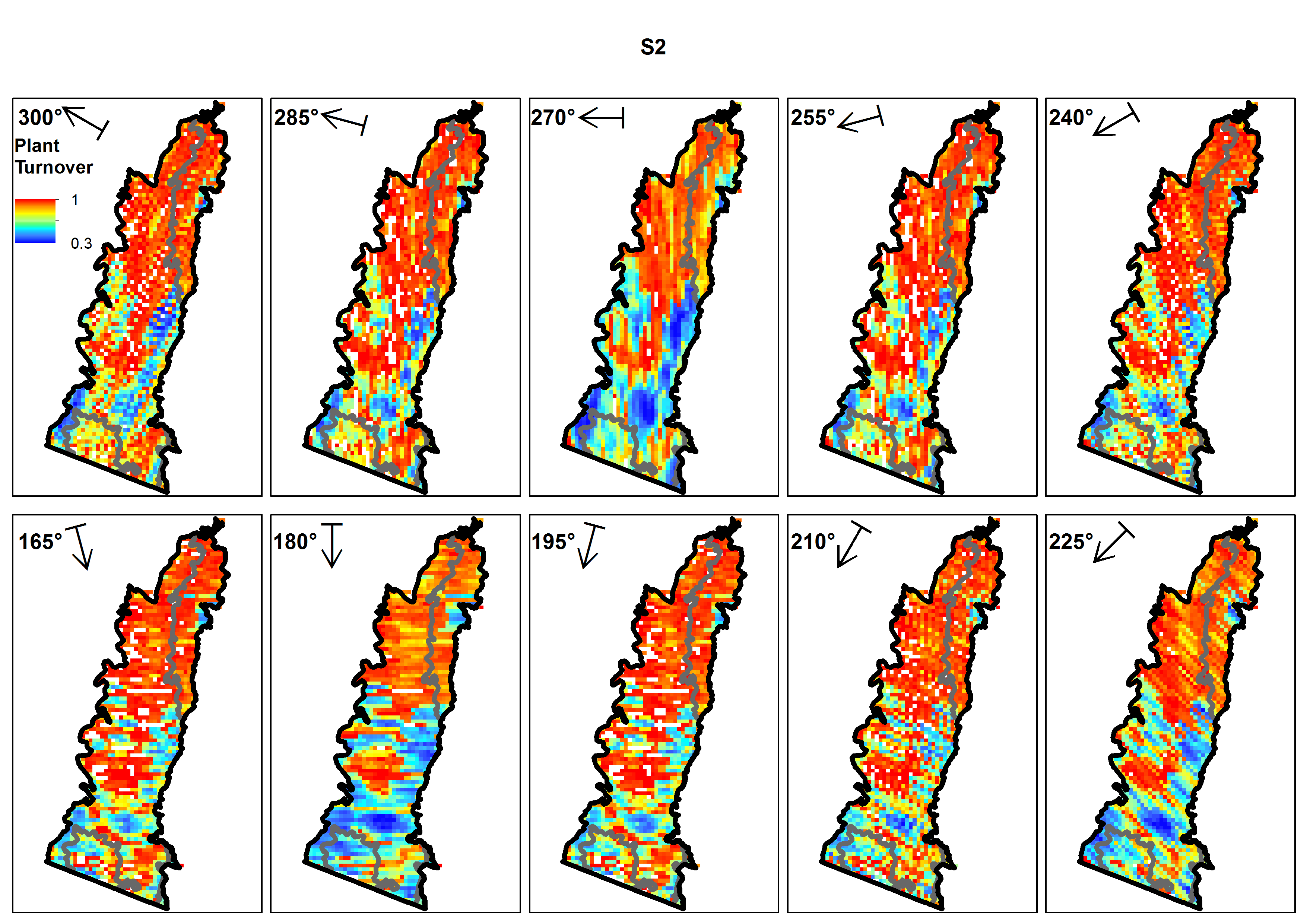

Supplement: Figure S2 — Plant species turnover maps in the South East Corner (SEC) bioregion of south-eastern New South Wales, Australia, for Sørensen moving window analyses rotated through 360° in 15° increments. Plant species turnover maps, moving window orientations 300° to 165°. (TIF) [file pone.0059227.s002.tif]

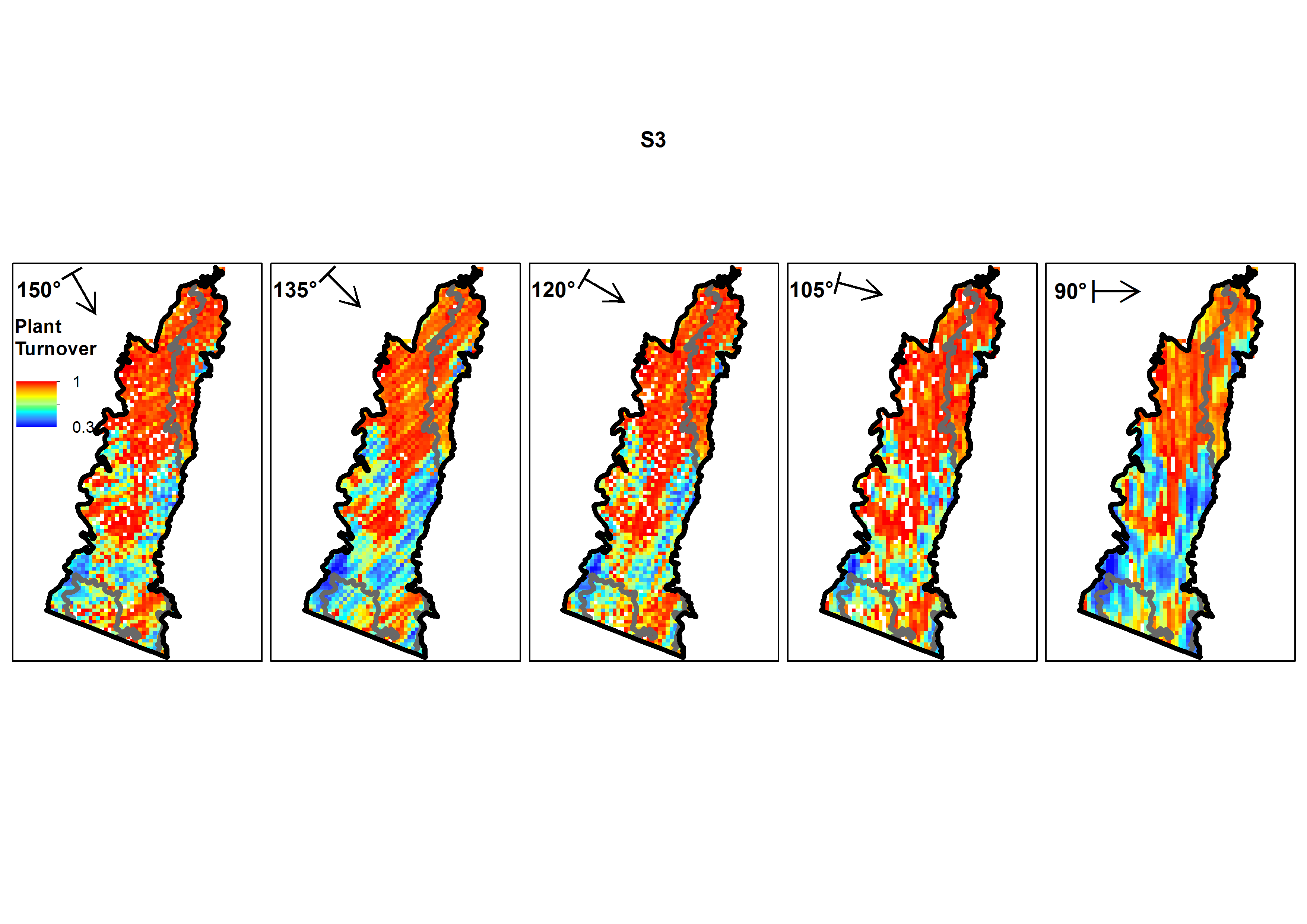

Supplement: Figure S3 — Plant species turnover maps in the South East Corner (SEC) bioregion of south-eastern New South Wales, Australia, for Sørensen moving window analyses rotated through 360° in 15° increments. Plant species turnover maps, moving window orientations 150° to 90°. (TIF) [file pone.0059227.s003.tif]
