# Supplementary material for: Quantifying High Resolution Transitional Breaks in Plant and Mammal Distributions at Regional Extent and Their Association with Climate, Topography and Geology
Source: PLoS One. 2013 Apr 1;8(4):e59227. doi: 10.1371/journal.pone.0059227 (PMC3613380; doi:10.1371/journal.pone.0059227)
Supplement: File S1 — Comparison of the plant and mammal genera unique to northern break and eastern overlap zones, unique to the same break and a western overlap zone and common to the intersections of each pair. Table S1, Plant genera that occur exclusively in the northern biogeographic break zone (n = 79), exclusively in the eastern overlap zone (n = 79) and genera that occur in both areas (n = 270) within the South East Corner (SEC) study area in New South Wales, Australia. Table S2, Plant genera that occur exclusively in the northern biogeographic break zone (n = 135), exclusively in the western overlap zone (n = 57) and genera that occur in both areas (n = 214) within the South East Corner (SEC) study area in New South Wales, Australia. Table S3, Mammal genera that occur exclusively in the northern biogeographic break zone (n = 2), exclusively in the eastern overlap zone (n = 1) and genera that occur in both areas (n = 32) within the South East Corner (SEC) study area in New South Wales, Australia. Table S4, Mammal genera that occur exclusively in the northern biogeographic break zone (n = 8), exclusively in the western overlap zone (n = 0) and genera that occur in both areas (n = 26) within the South East Corner (SEC) study area in New South Wales, Australia. (DOC) [file pone.0059227.s004.doc]

**Tables S1 – S4, File S1.** Comparison of the plant and mammal genera unique to northern break and eastern overlap zones, unique to the northern break and a western overlap zone and common to the intersections of each pair.

Table S1. Plant genera that occur exclusively in the northern biogeographic break zone (n = 79), exclusively in the eastern overlap zone (n = 79) and genera that occur in both areas (n = 270) within the South East Corner (SEC) study area in New South Wales, Australia.

| Both Neighbour Sets (List A Genera) | Neighbour Set 1 - Northern Break Zone (List B Genera) | Neighbour Set 2 - Eastern Overlap Zone (List C Genera) |
| --- | --- | --- |
| Acacia | Abutilon | Actinotus |
| Acaena | Achyranthes | Alphitonia |
| Acianthus | Acrothamnus | Ammobium |
| Acmena | Aldrovanda | Amperea |
| Acronychia | Alisma | Anisopogon |
| Acrotriche | Alternanthera | Apium |
| Actites | Aneilema | Arrhenechthites |
| Adiantum | Baumea | Arthropteris |
| Adriana | Boerhavia | Astroloma |
| Ajuga | Bolboschoenus | Bauera |
| Alectryon | Boronia | Brachychiton |
| Allocasuarina | Brachyscome | Brachyloma |
| Alyxia | Brasenia | Brunoniella |
| Amyema | Caesia | Burchardia |
| Angophora | Callicoma | Callitris |
| Aotus | Calochilus | Calocephalus |
| Aphanopetalum | Calytrix | Celastrus |
| Aristida | Cardamine | Cenchrus |
| Arthropodium | Casuarina | Centipeda |
| Asplenium | Caustis | Cephalomanes |
| Astrotricha | Ceratopetalum | Chiloglottis |
| Atriplex | Chamaesyce | Chorizema |
| Austrocynoglossum | Chloanthes | Convolvulus |
| Austrodanthonia | Choretrum | Cryptandra |
| Austrofestuca | Cladium | Cryptostylis |
| Austrostipa | Corybas | Daphnandra |
| Avicennia | Cuscuta | Darwinia |
| Backhousia | Cyathochaeta | Davallia |
| Banksia | Dendrophthoe | Deeringia |
| Bedfordia | Derwentia | Dennstaedtia |
| Bertya | Diplodium | Digitalis |
| Beyeria | Dryopoa | Diplazium |
| Billardiera | Elaeodendron | Disphyma |
| Blechnum | Enchylaena | Drosera |
| Bossiaea | Genoplesium | Empodisma |
| Bothriochloa | Glochidion | Erodium |
| Breynia | Glyceria | Eupomatia |
| Bulbine | Grammitis | Gleichenia |
| Bursaria | Halophila | Gnaphalium |
| Caladenia | Haloragis | Goodia |
| Callistemon | Haloragodendron | Histiopteris |
| Calochlaena | Hypoxis | Isachne |
| Calomeria | Jacksonia | Isotoma |
| Calystegia | Lachnagrostis | Lemna |
| Carex | Lepidium | Leptinella |
| Carpobrotus | Limosella | Leptocarpus |
| Cassinia | Lyperanthus | Leptomeria |
| Cassytha | Lythrum | Leptorhynchos |
| Centella | Marsilea | Lepyrodia |
| Centrolepis | Micrantheum | Livistona |
| Cestichis | Microtis | Lycopodiella |
| Cheilanthes | Mirbelia | Lysimachia |
| Chenopodium | Monotaxis | Maytenus |
| Chloris | Myriophyllum | Mitrasacme |
| Chrysocephalum | Najas | Passiflora |
| Claoxylon | Neopaxia | Phragmites |
| Clematis | Olax | Piper |
| Comesperma | Orthosiphon | Platycerium |
| Commelina | Oxylobium | Plectorrhiza |
| Conospermum | Paspalum | Ptilothrix |
| Coopernookia | Pentapogon | Restio |
| Coprosma | Picris | Ricinocarpos |
| Coronidium | Polygala | Ripogonum |
| Correa | Posidonia | Rumohra |
| Corymbia | Potamogeton | Scutellaria |
| Cotula | Prasophyllum | Selaginella |
| Craspedia | Pseudanthus | Sorghum |
| Crassula | Pseudoraphis | Sphaerolobium |
| Crepidomanes | Rostranthus | Stephania |
| Crowea | Scleria | Tetrarrhena |
| Cyathea | Sebaea | Teucrium |
| Cymbidium | Solenogyne | Thelionema |
| Cymbopogon | Spiranthes | Thysanotus |
| Cynodon | Styphelia | Tmesipteris |
| Cynoglossum | Thesium | Trophis |
| Cyperus | Todea | Urochloa |
| Dampiera | Tricostularia | Vernonia |
| Daucus | Viminaria | Wilkiea |
| Daviesia | Wolffia | Xerochrysum |
| Dendrobium |  |  |
| Desmodium |  |  |
| Deyeuxia |  |  |
| Dianella |  |  |
| Dichelachne |  |  |
| Dichondra |  |  |
| Dicksonia |  |  |
| Digitaria |  |  |
| Dillwynia |  |  |
| Dipodium |  |  |
| Distichlis |  |  |
| Diuris |  |  |
| Dodonaea |  |  |
| Doodia |  |  |
| Doryphora |  |  |
| Echinopogon |  |  |
| Ehretia |  |  |
| Einadia |  |  |
| Elaeocarpus |  |  |
| Eleocharis |  |  |
| Elymus |  |  |
| Entolasia |  |  |
| Epacris |  |  |
| Epilobium |  |  |
| Eragrostis |  |  |
| Eriochilus |  |  |
| Eucalyptus |  |  |
| Euchiton |  |  |
| Eucryphia |  |  |
| Eustrephus |  |  |
| Exocarpos |  |  |
| Ficinia |  |  |
| Ficus |  |  |
| Fieldia |  |  |
| Fimbristylis |  |  |
| Gahnia |  |  |
| Galium |  |  |
| Geitonoplesium |  |  |
| Geranium |  |  |
| Glossodia |  |  |
| Glycine |  |  |
| Gompholobium |  |  |
| Gonocarpus |  |  |
| Goodenia |  |  |
| Gratiola |  |  |
| Grevillea |  |  |
| Gynatrix |  |  |
| Hakea |  |  |
| Hardenbergia |  |  |
| Hedycarya |  |  |
| Helichrysum |  |  |
| Hibbertia |  |  |
| Hibiscus |  |  |
| Hierochloe |  |  |
| Homalanthus |  |  |
| Hovea |  |  |
| Howittia |  |  |
| Hydrocotyle |  |  |
| Hymenophyllum |  |  |
| Hypericum |  |  |
| Hypolepis |  |  |
| Imperata |  |  |
| Indigofera |  |  |
| Isolepis |  |  |
| Joycea |  |  |
| Juncus |  |  |
| Kennedia |  |  |
| Kunzea |  |  |
| Lagenophora |  |  |
| Lastreopsis |  |  |
| Leionema |  |  |
| Lepidosperma |  |  |
| Leptospermum |  |  |
| Leucopogon |  |  |
| Libertia |  |  |
| Limonium |  |  |
| Lindsaea |  |  |
| Lissanthe |  |  |
| Lobelia |  |  |
| Logania |  |  |
| Lomandra |  |  |
| Lomatia |  |  |
| Ludwigia |  |  |
| Luzula |  |  |
| Lycopodium |  |  |
| Marsdenia |  |  |
| Melaleuca |  |  |
| Mentha |  |  |
| Microlaena |  |  |
| Microsorum |  |  |
| Mimulus |  |  |
| Monotoca |  |  |
| Morinda |  |  |
| Muehlenbeckia |  |  |
| Muellerina |  |  |
| Myoporum |  |  |
| Myrsine |  |  |
| Notelaea |  |  |
| Notodanthonia |  |  |
| Olearia |  |  |
| Opercularia |  |  |
| Oplismenus |  |  |
| Oxalis |  |  |
| Ozothamnus |  |  |
| Pandorea |  |  |
| Panicum |  |  |
| Parsonsia |  |  |
| Paspalidium |  |  |
| Patersonia |  |  |
| Pelargonium |  |  |
| Pellaea |  |  |
| Persicaria |  |  |
| Persoonia |  |  |
| Philotheca |  |  |
| Phyllanthus |  |  |
| Pittosporum |  |  |
| Plantago |  |  |
| Platylobium |  |  |
| Platysace |  |  |
| Plectranthus |  |  |
| Poa |  |  |
| Podolobium |  |  |
| Polyscias |  |  |
| Polystichum |  |  |
| Pomaderris |  |  |
| Pomax |  |  |
| Poranthera |  |  |
| Pratia |  |  |
| Prostanthera |  |  |
| Pseuderanthemum |  |  |
| Pseudognaphalium |  |  |
| Psychotria |  |  |
| Pteridium |  |  |
| Pteris |  |  |
| Pterostylis |  |  |
| Pultenaea |  |  |
| Pyrrosia |  |  |
| Ranunculus |  |  |
| Rhagodia |  |  |
| Rhytidosporum |  |  |
| Rubus |  |  |
| Rumex |  |  |
| Ruppia |  |  |
| Sambucus |  |  |
| Samolus |  |  |
| Santalum |  |  |
| Sarcochilus |  |  |
| Sarcocornia |  |  |
| Sarcomelicope |  |  |
| Sarcopetalum |  |  |
| Scaevola |  |  |
| Schizomeria |  |  |
| Schoenoplectus |  |  |
| Schoenus |  |  |
| Sclerostegia |  |  |
| Selliera |  |  |
| Senecio |  |  |
| Senna |  |  |
| Sicyos |  |  |
| Sigesbeckia |  |  |
| Smilax |  |  |
| Solanum |  |  |
| Sparganium |  |  |
| Spinifex |  |  |
| Sporobolus |  |  |
| Spyridium |  |  |
| Stackhousia |  |  |
| Stellaria |  |  |
| Sticherus |  |  |
| Stypandra |  |  |
| Suaeda |  |  |
| Synoum |  |  |
| Tetragonia |  |  |
| Tetratheca |  |  |
| Themeda |  |  |
| Tricoryne |  |  |
| Triglochin |  |  |
| Tristaniopsis |  |  |
| Tylophora |  |  |
| Utricularia |  |  |
| Vallisneria |  |  |
| Veronica |  |  |
| Villarsia |  |  |
| Vittadinia |  |  |
| Wahlenbergia |  |  |
| Westringia |  |  |
| Wilsonia |  |  |
| Xanthosia |  |  |
| Zieria |  |  |
| Zornia |  |  |
| Zoysia |  |  |
| n= 270 | n= 79 | n= 79 |

Table S2. Plant genera that occur exclusively in the northern biogeographic break zone (n = 135), exclusively in the western overlap zone (n = 57) and genera that occur in both areas (n = 214) within the South East Corner (SEC) study area in New South Wales, Australia.

| Both Neighbour Sets (List A Genera) | Neighbour Set 1 - Northern Break Zone (List B Genera) | Neighbour Set 2 - Western Overlap Zone (List C Genera) |
| --- | --- | --- |
| Acacia | Abutilon | Amperea |
| Acaena | Achyranthes | Amphipogon |
| Acianthus | Acronychia | Arrhenechthites |
| Acmena | Acrothamnus | Arthropteris |
| Acrotriche | Actites | Asperula |
| Adiantum | Adriana | Astroloma |
| Ajuga | Aldrovanda | Baeckea |
| Alectryon | Alisma | Brachychiton |
| Allocasuarina | Alternanthera | Brachyloma |
| Amyema | Alyxia | Calandrinia |
| Angophora | Aneilema | Callitris |
| Aphanopetalum | Aotus | Calotis |
| Aristida | Astrotricha | Capillipedium |
| Arthropodium | Atriplex | Celastrus |
| Asplenium | Austrofestuca | Cenchrus |
| Austrocynoglossum | Austrostipa | Cleistochloa |
| Austrodanthonia | Avicennia | Convolvulus |
| Backhousia | Baumea | Cryptandra |
| Banksia | Bertya | Davallia |
| Bedfordia | Boerhavia | Deeringia |
| Beyeria | Bolboschoenus | Dennstaedtia |
| Billardiera | Bothriochloa | Diplarrena |
| Blechnum | Brasenia | Diplazium |
| Boronia | Bulbine | Drosera |
| Bossiaea | Caesia | Drymophila |
| Brachyscome | Caladenia | Empodisma |
| Breynia | Callicoma | Eriostemon |
| Bursaria | Calochilus | Eupomatia |
| Callistemon | Calytrix | Euryomyrtus |
| Calochlaena | Cardamine | Gaultheria |
| Calomeria | Carpobrotus | Goodia |
| Calystegia | Casuarina | Histiopteris |
| Carex | Centella | Isopogon |
| Cassinia | Centrolepis | Isotoma |
| Cassytha | Ceratopetalum | Laxmannia |
| Caustis | Cestichis | Leptinella |
| Cheilanthes | Chamaesyce | Leptomeria |
| Choretrum | Chenopodium | Lepyrodia |
| Claoxylon | Chloanthes | Lotus |
| Clematis | Chloris | Micromyrtus |
| Comesperma | Chrysocephalum | Mitrasacme |
| Commelina | Cladium | Nematolepis |
| Coopernookia | Conospermum | Nertera |
| Coprosma | Corybas | Nestegis |
| Coronidium | Corymbia | Omphacomeria |
| Correa | Cotula | Oreomyrrhis |
| Crassula | Craspedia | Passiflora |
| Crepidomanes | Cuscuta | Phyllota |
| Crowea | Cyathochaeta | Sorghum |
| Cyathea | Cynodon | Stephania |
| Cymbidium | Dendrophthoe | Tmesipteris |
| Cymbopogon | Distichlis | Trachymene |
| Cynoglossum | Dryopoa | Uncinia |
| Cyperus | Ehretia | Velleia |
| Dampiera | Elaeodendron | Vernonia |
| Daucus | Eleocharis | Xerochrysum |
| Daviesia | Elymus | Youngia |
| Dendrobium | Enchylaena |  |
| Derwentia | Epilobium |  |
| Desmodium | Eriochilus |  |
| Deyeuxia | Ficinia |  |
| Dianella | Fimbristylis |  |
| Dichelachne | Genoplesium |  |
| Dichondra | Glochidion |  |
| Dicksonia | Glossodia |  |
| Digitaria | Glyceria |  |
| Dillwynia | Gynatrix |  |
| Diplodium | Halophila |  |
| Dipodium | Haloragis |  |
| Diuris | Helichrysum |  |
| Dodonaea | Hibiscus |  |
| Doodia | Howittia |  |
| Doryphora | Hypoxis |  |
| Echinopogon | Lepidium |  |
| Einadia | Libertia |  |
| Elaeocarpus | Limonium |  |
| Entolasia | Limosella |  |
| Epacris | Lissanthe |  |
| Eragrostis | Lobelia |  |
| Eucalyptus | Ludwigia |  |
| Euchiton | Lyperanthus |  |
| Eucryphia | Lythrum |  |
| Eustrephus | Micrantheum |  |
| Exocarpos | Microlaena |  |
| Ficus | Microtis |  |
| Fieldia | Mimulus |  |
| Gahnia | Myriophyllum |  |
| Galium | Najas |  |
| Geitonoplesium | Neopaxia |  |
| Geranium | Olax |  |
| Glycine | Orthosiphon |  |
| Gompholobium | Paspalum |  |
| Gonocarpus | Pelargonium |  |
| Goodenia | Pentapogon |  |
| Grammitis | Picris |  |
| Gratiola | Platylobium |  |
| Grevillea | Posidonia |  |
| Hakea | Potamogeton |  |
| Haloragodendron | Prasophyllum |  |
| Hardenbergia | Pseudanthus |  |
| Hedycarya | Pseuderanthemum |  |
| Hibbertia | Pseudoraphis |  |
| Hierochloe | Psychotria |  |
| Homalanthus | Rhagodia |  |
| Hovea | Rostranthus |  |
| Hydrocotyle | Ruppia |  |
| Hymenophyllum | Samolus |  |
| Hypericum | Sarcocornia |  |
| Hypolepis | Sarcomelicope |  |
| Imperata | Schizomeria |  |
| Indigofera | Schoenoplectus |  |
| Isolepis | Scleria |  |
| Jacksonia | Sclerostegia |  |
| Joycea | Sebaea |  |
| Juncus | Selliera |  |
| Kennedia | Senna |  |
| Kunzea | Solenogyne |  |
| Lachnagrostis | Sparganium |  |
| Lagenophora | Spinifex |  |
| Lastreopsis | Spiranthes |  |
| Leionema | Sporobolus |  |
| Lepidosperma | Suaeda |  |
| Leptospermum | Synoum |  |
| Leucopogon | Tetragonia |  |
| Lindsaea | Todea |  |
| Logania | Tricostularia |  |
| Lomandra | Triglochin |  |
| Lomatia | Utricularia |  |
| Luzula | Vallisneria |  |
| Lycopodium | Villarsia |  |
| Marsdenia | Viminaria |  |
| Marsilea | Westringia |  |
| Melaleuca | Wilsonia |  |
| Mentha | Wolffia |  |
| Microsorum | Zoysia |  |
| Mirbelia |  |  |
| Monotaxis |  |  |
| Monotoca |  |  |
| Morinda |  |  |
| Muehlenbeckia |  |  |
| Muellerina |  |  |
| Myoporum |  |  |
| Myrsine |  |  |
| Notelaea |  |  |
| Notodanthonia |  |  |
| Olearia |  |  |
| Opercularia |  |  |
| Oplismenus |  |  |
| Oxalis |  |  |
| Oxylobium |  |  |
| Ozothamnus |  |  |
| Pandorea |  |  |
| Panicum |  |  |
| Parsonsia |  |  |
| Paspalidium |  |  |
| Patersonia |  |  |
| Pellaea |  |  |
| Persicaria |  |  |
| Persoonia |  |  |
| Philotheca |  |  |
| Phyllanthus |  |  |
| Pittosporum |  |  |
| Plantago |  |  |
| Platysace |  |  |
| Plectranthus |  |  |
| Poa |  |  |
| Podolobium |  |  |
| Polygala |  |  |
| Polyscias |  |  |
| Polystichum |  |  |
| Pomaderris |  |  |
| Pomax |  |  |
| Poranthera |  |  |
| Pratia |  |  |
| Prostanthera |  |  |
| Pseudognaphalium |  |  |
| Pteridium |  |  |
| Pteris |  |  |
| Pterostylis |  |  |
| Pultenaea |  |  |
| Pyrrosia |  |  |
| Ranunculus |  |  |
| Rhytidosporum |  |  |
| Rubus |  |  |
| Rumex |  |  |
| Sambucus |  |  |
| Santalum |  |  |
| Sarcochilus |  |  |
| Sarcopetalum |  |  |
| Scaevola |  |  |
| Schoenus |  |  |
| Senecio |  |  |
| Sicyos |  |  |
| Sigesbeckia |  |  |
| Smilax |  |  |
| Solanum |  |  |
| Spyridium |  |  |
| Stackhousia |  |  |
| Stellaria |  |  |
| Sticherus |  |  |
| Stypandra |  |  |
| Styphelia |  |  |
| Tetratheca |  |  |
| Themeda |  |  |
| Thesium |  |  |
| Tricoryne |  |  |
| Tristaniopsis |  |  |
| Tylophora |  |  |
| Veronica |  |  |
| Vittadinia |  |  |
| Wahlenbergia |  |  |
| Xanthosia |  |  |
| Zieria |  |  |
| Zornia |  |  |
| n= 214 | n= 135 | n= 57 |

Table S3. Mammal genera that occur exclusively in the northern biogeographic break zone (n = 2), exclusively in the eastern overlap zone (n = 1) and genera that occur in both areas (n = 32) within the South East Corner (SEC) study area in New South Wales, Australia.

| Both Neighbour Sets (List A Genera) | Neighbour Set 1 - Northern Break Zone (List B Genera) | Neighbour Set 2 - Eastern Overlap Zone (List C Genera) |
| --- | --- | --- |
| Acrobates | Myotis | Isoodon |
| Antechinus | Saccolaimus |  |
| Cercartetus |  |  |
| Chalinolobus |  |  |
| Dasyurus |  |  |
| Falsistrellus |  |  |
| Hydromys |  |  |
| Kerivoula |  |  |
| Macropus |  |  |
| Miniopterus |  |  |
| Mormopterus |  |  |
| Nyctophilus |  |  |
| Ornithorhynchus |  |  |
| Perameles |  |  |
| Petauroides |  |  |
| Petaurus |  |  |
| Phascogale |  |  |
| Phascolarctos |  |  |
| Potorous |  |  |
| Pseudocheirus |  |  |
| Pteropus |  |  |
| Rattus |  |  |
| Rhinolophus |  |  |
| Scoteanax |  |  |
| Scotorepens |  |  |
| Sminthopsis |  |  |
| Tachyglossus |  |  |
| Tadarida |  |  |
| Trichosurus |  |  |
| Vespadelus |  |  |
| Vombatus |  |  |
| Wallabia |  |  |
| n= 32 | n= 2 | n = 1 |

Table S4. Mammal genera that occur exclusively in the northern biogeographic break zone (n = 8), exclusively in the western overlap zone (n = 0) and genera that occur in both areas (n = 26) within the South East Corner (SEC) study area in New South Wales, Australia.

| Both Neighbour Sets (List A Genera) | Neighbour Set 1 - Northern Break Zone (List B Genera) | Neighbour Set 2 - Western Overlap Zone (List C Genera) |
| --- | --- | --- |
| Acrobates | Cercartetus | No records |
| Antechinus | Kerivoula |  |
| Chalinolobus | Miniopterus |  |
| Dasyurus | Mormopterus |  |
| Falsistrellus | Potorous |  |
| Hydromys | Rhinolophus |  |
| Macropus | Saccolaimus |  |
| Myotis | Sminthopsis |  |
| Nyctophilus |  |  |
| Ornithorhynchus |  |  |
| Perameles |  |  |
| Petauroides |  |  |
| Petaurus |  |  |
| Phascogale |  |  |
| Phascolarctos |  |  |
| Pseudocheirus |  |  |
| Pteropus |  |  |
| Rattus |  |  |
| Scoteanax |  |  |
| Scotorepens |  |  |
| Tachyglossus |  |  |
| Tadarida |  |  |
| Trichosurus |  |  |
| Vespadelus |  |  |
| Vombatus |  |  |
| Wallabia |  |  |
| n= 26 | n= 8 | n= 0 |
